# Selecting Wearable Devices to Measure Cardiovascular Functions in Community-Dwelling Adults: Application of a Practical Guide for Device Selection

**DOI:** 10.1016/j.mcpdig.2025.100202

**Published:** 2025-03-12

**Authors:** Jessica K. Lu, Weilan Wang, Jorming Goh, Andrea B. Maier

**Affiliations:** a Healthy Longevity Translational Research Programme, Yong Loo Lin School of Medicine, National University of Singapore, Singapore, Singapore; b NUS Academy for Healthy Longevity, Yong Loo Lin School of Medicine, National University of Singapore, Singapore, Singapore; c Centre for Healthy Longevity, National University Health System, Singapore, Singapore; d Department of Physiology, Yong Loo Lin School of Medicine, National University of Singapore, Singapore, Singapore; e Department of Human Movement Sciences, @AgeAmsterdam, Faculty of Behavioural and Movement Sciences, Vrije Universiteit Amsterdam, Amsterdam Movement Sciences, Amsterdam, Netherlands

## Abstract

Continuous monitoring of cardiovascular functions can provide crucial insights into the health status and lifestyle behaviors of an individual. Wearable devices offer a convenient and cost-effective solution for collecting cardiovascular measurements outside clinical settings. However, the abundance of available devices poses challenges for researchers, health care professionals, and device users in selecting the most suitable one. This article illustrates the application of a practical guide for selecting wearable devices for the continuous monitoring of cardiovascular functions in community-dwelling adults who are generally healthy or have minimal, well-managed chronic conditions. An initial systematic review of the literature revealed 216 devices, each of which were assessed on the basis of 5 core criteria from the guide: (1) continuous monitoring capability, (2) device availability and suitability, (3) technical performance (accuracy and precision), (4) feasibility of use, and (5) cost evaluation. From the 216 devices, there were 136 devices capable of continuous monitoring. After the exclusion of unavailable and unsuitable devices, 53 devices underwent validation assessment of accuracy and precision. Although COSMIN criteria were applied to evaluate technical performance, a lack of validation for certain devices limits a comprehensive evaluation. After selection of valid devices, the feasibility and cost of 20 devices were examined. Wearable devices, such as the Apple Watch Series 9, Fitbit Charge 6, Garmin vívosmart 5, and Oura Ring Gen3, emerged as suitable devices to measure cardiovascular function in community-dwelling adults. The systematic process for device selection could also be applied to select wearable devices for the measurement of other physiologic variables and lifestyle behaviors.

**Article Highlights**

• This study illustrated the application of a practical guide with 5 core criteria for selecting wearable devices in clinical research and practice.
• It evaluated over 200 wearable devices for cardiovascular monitoring, narrowing them down to 20 suitable options on the basis of accuracy, usability, and practicality.
• It emphasized user comfort, wearability, and integration into clinical workflows to ensure feasibility and adherence in community-dwelling adults in real-world settings.

Continuous monitoring of physiologic variables offers valuable insights into an individual’s health status influenced by their lifestyle behaviors. Among these variables, cardiovascular measurements play an important role in health status assessments and determining the effects of clinical interventions.^1^ Metrics like heart rate (HR), heart rate variability (HRV), and blood pressure (BP) offer indicators of cardiovascular function,^2^ which can be effectively tracked by wearable devices, such as activity trackers and smartwatches.^3^ Wearable devices, with their ability to monitor cardiovascular functions continuously, noninvasively, and remotely,^4^^,^^5^ have become convenient and cost-effective solutions in clinical practice and research to supplement traditional periodical clinic visits,^6^ which may not capture day-to-day variations.^7^

The array of wearable devices for cardiovascular monitoring has expanded exponentially, and selecting the most suitable device remains a complex task. Existing guidelines often provide theoretical frameworks for selection but lack step-by-step guidance for practical implementation. The practical guide used in this study provides a systematic, actionable approach to streamline device selection. It includes 5 core criteria: (1) continuous monitoring capability, (2) device availability and suitability, (3) technical performance (accuracy and precision), (4) feasibility of use, and (5) cost evaluation.^8^ Device availability ensures the device can be easily obtained by researchers, health care professionals (HCPs), and device users, rather than being limited to prototypes or research-only models. Device suitability considers the logistics and user burden of using the device daily. Accuracy is vital to ensure reliable measurements,^3^ whereas feasibility^2^ and cost-effectiveness^4^ of these devices influence the degree of acceptance and adoption of wearable devices for consistent usage, facilitating regular use of the device with minimal interruptions to allow for reliable data collection over time. Feasibility is important to ensure that devices are practical and usable for community-dwelling adults, enabling adherence during unsupervised use.

The objective of this study was to use the practical guide to select wearable devices capable of continuous monitoring to support the long-term collection of cardiovascular data in community-dwelling adults who are generally healthy or have minimal, well-managed chronic conditions. These criteria from the practical guide are central to achieving the study’s objective of providing actionable guidance for researchers and HCPs during device selection.

## Methods

### Search Strategy and Selection Criteria

A post hoc analysis of a systematic review, which described 216 distinct devices for measuring cardiovascular functions in community-dwelling individuals, was performed to select suitable noninvasive devices that can be used unsupervised.^9^ This means that the devices are user-friendly and operable independently by adults without requiring oversight by a HCP or other trained personnel. To select a suitable device, a practical guide comprising the following 5 core criteria was used: (1) continuous monitoring capability, (2) device availability and suitability, (3) technical performance (accuracy and precision), (4) feasibility of use, and (5) cost evaluation.^8^

### Criterion 1: Continuous Monitoring Capability

This criterion evaluated whether the device can passively (ie, without user interaction) collect measurements for 24 hours a day for a minimum of 7 days. Continuous monitoring capability was prioritized to ensure that devices can collect data over extended periods without interruption, a key requirement for long-term, unsupervised use in research and clinical applications. The cardiovascular variables measured include BP, HR, HRV, and blood oxygenation (SpO_2_). Devices that do not facilitate continuous monitoring were excluded.

### Criterion 2: Device Availability and Suitability

This criterion considered whether a device was readily accessible and suitable for use by researchers, HCPs, or device users. Devices were excluded if the model was discontinued, if it was a prototype with limited validation, or if it lacked support for up-to-date hardware and software.^10^^,^^11^ Outdated models were replaced with the latest model as of July 1, 2024, from the original manufacturer. Given the rapid pace of technological development, selecting devices that are current at the time of evaluation ensures compatibility, longevity, and practical applicability in both research and clinical settings. Devices were excluded if they were considered unsuitable for use in healthy adults. This includes devices that were (i) bulky or obtrusive, making them impractical for daily activities, such as 24-hour ambulatory BP monitors, Holter electrocardiograms (ECGs) with a crossbody recorder, wearable cardioverter defibrillators, and wrist pulse oximeters with finger attachments that would obstruct the hands, arms, legs, or feet of the user during activities of daily living; and/or (ii) designed for specific medical indications irrelevant to this study population, such as pregnancy or sleep apnea monitoring.^12^ These exclusion criteria ensured that selected devices were practical, minimally invasive, and aligned with the focus on continuous monitoring in healthy individuals.

### Criterion 3: Technical Performance (Accuracy and Precision)

Devices were evaluated on their accuracy (measurement error) and precision (reliability) to measure cardiovascular variables. Reporting of verification and validation conducted in community-dwelling adults was first searched in the articles included in the systematic review,^9^ then searched from the references cited in the included articles. If no data could be obtained from these sources, a search was conducted on PubMed until July 1, 2024, using search terms related to “validation” and “[device brand and model]” (eg, Garmin vívosmart), and the website of the brand was searched up till July 1, 2024, for validation articles.^9^ Validation measures for accuracy included standard error of measurement, limits of agreement, or coefficient of variation. Validation measures for precision included intraclass correlation coefficient (ICC), Lin's concordance correlation coefficient, Pearson correlation coefficient (*r*), or Spearman correlation coefficient (ρ). Adapted from the COnsensus-based Standards for the selection of health Measurement INstruments (COSMIN) Risk of Bias tool,^13^ sufficient accuracy was defined as (limits of agreement or 95% CI of the coefficient of variation) < Minimal Important Change or determined using the conclusions from the validation studies. Sufficient precision was defined as ICC or (weighted) κ≥0.70.^13^ Validation results were reported for BP, HR, HRV, and SpO_2_ (and their variations of measurement, ie, R-R interval or root mean square of successive differences for HRV). For devices measuring BP, devices were considered accurate and precise if the measurements met the clinically accepted requirements of the US Association for the Advancement of Medical Instrumentation/European Society of Hypertension/International Organization for Standardization or the British Hypertension Society. If validation studies reported results of measurement accuracy and precision during rest and during activity/exercise, both were reported in Appendix 1 (available online at https://www.mcpdigitalhealth.org/). Only validation results for when the device user is at rest were assessed as various types and intensities of activity/exercise were reported across the studies (Table 1).^14^, ^15^, ^16^, ^17^, ^18^, ^19^, ^20^, ^21^, ^22^, ^23^, ^24^, ^25^, ^26^, ^27^, ^28^, ^29^, ^30^, ^31^, ^32^, ^33^, ^34^ Devices were excluded if validation studies did not use a gold or reference standard or if measurement performance was not reported according to the COSMIN Risk of Bias tool^13^ or international clinically accepted guidelines. In cases where no gold standard was available, validation against a reliable clinical standard was considered acceptable. Devices were also excluded if validation studies reported indeterminate or inaccurate accuracy results^13^ or if no validation data were available in peer-reviewed articles or on manufacturer websites. This approach ensures that only devices with adequate validation are included, maintaining the reliability of the data collected.

**Table 1:** Selection of the 20 Wearable Devices for Measuring Cardiovascular Function That Can Be Used for Continuous Monitoring in Community-Dwelling Adults and Have Valid Measurements

Remote monitoring device	Current model (if applicable)	Accuracy (measurement error)	Precision (reliability)	Validation conclusion
		At rest	At rest	
Aktiia Bracelet	—	Bias±SEm^14^ SBP, 0.46±7.75 DBP, 0.39±6.86	PCC^14^ SBP, *r*=0.92 DBP, *r*=0.83	Sufficiently accurate and precise
Apple Watch Series 6	Apple Watch Series 9	Bias (LoA)^15^ HR, −0.11 (−5.84, 5.63) SpO_2_, −0.23 (−3.49, 3.04)	PCC^15^ HR, *r*=0.98 SpO_2_, *r*=0.89	Sufficiently accurate and precise
Biobeat Wrist Monitor	—	Bias (LoA)^16^ SBP, −0.08 (−7.06, 6.90) DBP, 0.00 (−6.88, 6.87)	ICC^16^ SBP, 0.99 DBP, 0.98	Sufficiently accurate and precise
Biovotion Everion bracelet	Biofourmis Everion+™	Bias (LoA)^17^ HR, −1.06 (−9.59, 7.47) HRV, −0.91 (−7.35, 5.53)	ICC (95% CI)^17^ HR, 0.97 (0.97, 0.98) HRV, 0.94 (0.90, 0.96)	Sufficiently accurate and precise
CardiacSense Wristwatch	—	Bias (LoA)^18^ RRi, −0.1 (−29.2, 29)	PCC^18^ RRi, *r*=0.99	Sufficiently accurate and precise
Corsano Cardiowatch 287 Bracelet	—	Bias (LoA)^19^ SBP, −0.17 (−8.74, 8.4) DBP, 0.2 (−6.96, 7.37) Bias (LoA)^20^ HR, −0.06 (−3.89, 3.77) RRi, −1 (−173, 171)	PCC^19^ SBP, *r*=0.985 DBP, *r*=0.961 PCC (95% CI)^20^ HR, *r*=0.99 (0.99, 0.99) RRi, *r*=0.89 (0.89, 0.90)	Sufficiently accurate and precise
Firstbeat Bodyguard 2 HR Monitor	Firstbeat Bodyguard 3	Bias^21^ RRi, −0.70 HRV (rMSSD), 7.99	—	Sufficient accuracy, indeterminate precision
Firstbeat Textile ECG Strap	—	Bias^22^ (LoA) HR, 0 (0, 0) HRV, −7.37 (−33.00, 18.27)	CCC^22^ HRV (rMSSD), 0.76	Sufficiently accurate and precise
Fitbit Charge 4	Fitbit Charge 6	Charge 2 Bias (LoA)^23^ HR, −1.26 (−12.4, 9.90) Charge 4 Bias (LoA)^24^ HR, 3.36 (−18.98, 25.70)	Charge 2 CCC (95% CI)^23^ HR, 0.89 (0.84, 0.92) Charge 4 CCC^24^ HR, 0.203 PCC HR, 0.257	Sufficiently accurate, but not precise
Garmin vívosmart 4	Garmin vívosmart 5	Bias (95% CI)^25^ HR, 1.76 (−19.98, 23.50)	ICC^25^ HR, 0.76	Sufficiently accurate and precise
Hexoskin Smart Shirt	—	CV±SD (%)^26^ HR, 0.52±0.55	ICC^26^ HR, 0.99	Sufficiently accurate and precise
LiveMetric LiveOne	—	Bias (LoA)^27^ SBP, 0.2 (−20.0, 20.4) DBP, 0.9 (−14.1, 15.9)	PCC^27^ SBP, *r*=0.91 DBP, *r*=0.85	Sufficiently accurate and precise
Omron HEM-6410T “HeartGuide”	—	Bias±SD^28^ SBP, 3.4±16.4 DBP, −3.2±11.1 PR, −0.1±6.1	PCC^28^ SBP, *r*=0.63 DBP, *r*=0.64 PR, *r*=0.87	BP measurements are insufficiently precise, but sufficiently accurate. PR measurements are accurate and precise
Oura Ring Gen2	Oura Ring Gen3	Bias (95% CI)^29^ HR, −0.63 (−1.38, 0.11) rMSSD, −1.2 (−8.8, 6.5) Bias (LoA)^22^ rMSSD, −2.33 (−17.85, 13.19)	PCC^29^ HR, 0.99 rMSSD, 0.98 CCC^22^ rMSSD, 0.91	Sufficiently accurate and precise
Polar H10 HR Sensor	—	Bias (LoA)^22^ HR, −0.32 (−1.92, 1.29) rMSSD, −6.98 (−34.74, 20.78)	CCC^22^ rMSSD, 0.77	Sufficiently accurate and precise
Polar Vantage M Watch	Polar Vantage M2	SEm^30^ HR, 8.71	ICC (95% CI)^30^ HR, 0.42 (0.27, 0.73)	Accurate, but not precise Authors say not acceptable for clinical use^30^
Samsung Galaxy Watch3 (SM-R850)	Samsung Galaxy Watch7	Bias (LoA)^24^ HR, 4.41 (−40.5, 49.33) Bias (LoA)^31^ SBP, 0.4 (−8.8, 9.2) DBP, 1.1 (−7.9, 10.1)	CCC^24^ HR, 0.51 PCC^24^ HR, *r*=0.56 SBP, *r*=0.97^31^ DBP, *r*=0.92^31^	Not sufficiently accurate or precise for HR measurements; sufficiently accurate and precise for BP measurements
VitalConnect VitalPatch RTM	—	Bias (LoA)^32^ HR, 0.31 (−3.8, 4.42)	PCC^32^ HR, *r*=0.99	Sufficiently accurate and precise
WHOOP Strap 2.0	WHOOP 4.0 Band	Bias (LoA)^33^ HR, −0.15 (−2.15, 1.85) HRV, 4.8 (−7.8, 17.4)	—	Sufficient accuracy, indeterminate precision
Zephyr BioPatch HP	—	Bias (95% CI)^34^ HR, −0.01 (−0.12, 0.10)	PCC^34^ HR, *r*=0.99	Sufficiently accurate and precise

Reference standard refers to the gold standard or clinical standard of measurement. Unless otherwise stated, BP is in units of mmHg, HR in bpm, HRV (rMMSD) in ms, RRi in ms, and SpO_2_ in %.

bpm, beats per minute; DBP, diastolic blood pressure; ECG, electrocardiogram; HR, heart rate; HRV, heart rate variability; LoA, 95% limits of agreement; PCC, Pearson correlation coefficient, *r*; rMSSD, root mean square of successive differences; RRi, R-R interval; SBP, systolic blood pressure; SEm, standard error of measurement; —, data not applicable or not reported.

### Criterion 4: Feasibility of Use

Feasibility relating to user experience (measurements validated, population validated, acceptability, usability, and battery life) and usage in research and/or clinical practice (device setup/maintenance, data management, firmware/software updates, and data access) was evaluated.^8^ Feasibility was included to assess the practicality of using these devices in community-dwelling populations, focusing on ease of use, wearability, and user adherence. The parameters in the “Usage in research and/or clinical practice” category help to assess the logistical and practical challenges of incorporating devices into research or clinical settings. Each feasibility parameter was categorized into high, medium, or low feasibility for using the device (Table 2).^8^ For population validated, the premise is that the population of interest is community-dwelling adults who are generally healthy or have minimal, well-managed chronic conditions, so it is more ideal to have the device be validated in healthy adults. To ensure consistent monitoring, devices were also evaluated on the basis of their practicality for daily use, including features that minimize user burden (eg, comfort, usability, and battery life) and reduce the likelihood of interruptions in data collection. Devices with poor adherence potential, such as requiring frequent user interaction or being easily removed during routine activities, were considered less suitable for consistent monitoring. Acceptability encompasses considerations of user burden, wearability, and comfort, focusing on how well the device integrates into the user’s daily life without causing physical or psychological discomfort. Usability includes adherence and long-term adherence, assessing the practicality of wearing the device over extended periods. These factors ensure that continuous monitoring remains realistic and effective in real-world settings. To assess the advantages and disadvantages of each device, information for each parameter was obtained via the website of the brand/product (as of July 1, 2024), from the included articles that reported the usage of the devices, and then the manufacturer was contacted for more information and specifications (Appendix 2, available online at https://www.mcpdigitalhealth.org/). The manufacturers were contacted via email or the webform on their websites with 2 follow-up emails/webforms after 1 week if no response was received after 1 week. Manufacturers contacted via email or webform are listed in Appendix 3 (available online at https://www.mcpdigitalhealth.org/). Data access and management, including the transparency of data processing algorithms and means of feedback (eg, directly to users or via a platform), were evaluated under feasibility to address integration into research or clinical workflows. When evaluating data access and management parameters, the interoperability of the devices with integration standards was also considered, including their ability to export data in versatile formats and integrate with commonly used clinical data management systems (eg, REDCap and IQVIA) and electronic health record or electronic medical record systems (eg, Epic and Cerner). Privacy and data security were also evaluated indirectly through these parameters, highlighting adaptation of workflows to account for device-specific challenges. Although specific workflows depend on the researchers or clinical team, these considerations provide a foundation for adapting the guide to diverse use cases.

**TABLE 2:** Feasibility Assessment to Determine Whether a Wearable Device Would Be Suitable for Use in Community-Dwelling Adults in Research and/or Clinical Practice

	Parameter			
User experience	Measurements validated (BP, HR, HRV, SpO_2_)	3 or more measurements	2 measurements	1 measurement
	Population validated	Healthy adults	Adults with diseases	Unknown/not reported
	Acceptability	Low discomfort (eg, finger/wrist-worn)	Medium discomfort (eg, chest straps, and arm bands)	High discomfort (eg, obtrusive and skin irritations)
	Usability (wear, usage, maintenance)	Device is water resistant and/or user only needs to wear the device and remove it for hygiene purposes (little to no interactions with user)	User needs to manually sync data (or confirm that data has been synced) but does not need to charge the device frequently	User needs to charge the device daily/clean the device regularly or another device is required for readout and/or upload of collected data
	Battery life (after 1 charge)	>7 d	3-7 d	<3 d; unknown/no information
Usage in research and/or clinical practice	Device setup/maintenance	Water resistant and device can be reused between different users; measurements collected and sampling frequency modifiable	Not water resistant, but device can be reused between different users after sanitizing	Device is not water resistant/cannot be sanitized between users (only 1 user per device); unknown/no information
	Data management	Device manufacturer provides a user-facing application for data syncing and researcher-based/HCP-facing online/cloud-based management platform. Moreover, functionality to provide feedback for users can be toggled on and off	Only a user-facing application and/or only a researcher-based/HCP-facing online/cloud-based management platform is available for uploading collected data	An external application/third-party software required to extract/export data collected by the device; unknown/no information
	Firmware/software updates	Researcher/HCP has control over whether to update the device firmware or data management platform software	Manufacturer is transparent with when updates are rolled out, but researcher/HCP has no control on whether to implement updates	No control over device firmware and software updates (automatic); unknown/no information
	Data access	Researcher/HCP have access to the raw data and data processing algorithms	Researcher/HCP can export only raw data in various formats, such as .csv and .json	Researcher/HCP cannot export (raw) data or device manufacturer has access to user data

Usage in research and/or clinical practice: , high feasibility; , medium feasibility; , low feasibility.

BP, blood pressure; HCP, health care professional; HR, heart rate; HRV, heart rate variability; SpO_2_, blood oxygen saturation.

Finally, the feasibility of using the device in clinical research was summarized, for which high, medium, or low feasibility were categorized as green, yellow, and red circles, respectively. Ideally, a greater number of green (high feasibility) and fewer number of red circles (low feasibility) are desired.

### Criterion 5: Cost Evaluation

Costs for the devices and related management systems and subscriptions were considered for this criterion. Cost cutoffs were not predefined to allow for flexibility on the basis of user-specific budgetary constraints, including device and accessory costs. The information was obtained via the website for the device, or the manufacturer was contacted for more information following the method described previously. The cost per device was current as of July 1, 2024.

## Results

### Criterion 1: Continuous Monitoring Capability

Of 216 devices for measuring cardiovascular function,^9^ 136 devices could be used for continuous monitoring (Figure; Appendix 1, available online at https://www.mcpdigitalhealth.org/).

### Criterion 2: Device Availability and Suitability

After removal of prototype, discontinued, bulky, and/or medical devices unsuitable for monitoring in healthy community-dwelling adults, 53 devices remained for further evaluation (Figure). This list of 53 devices includes the latest models that replaced the outdated ones.

**Figure:**
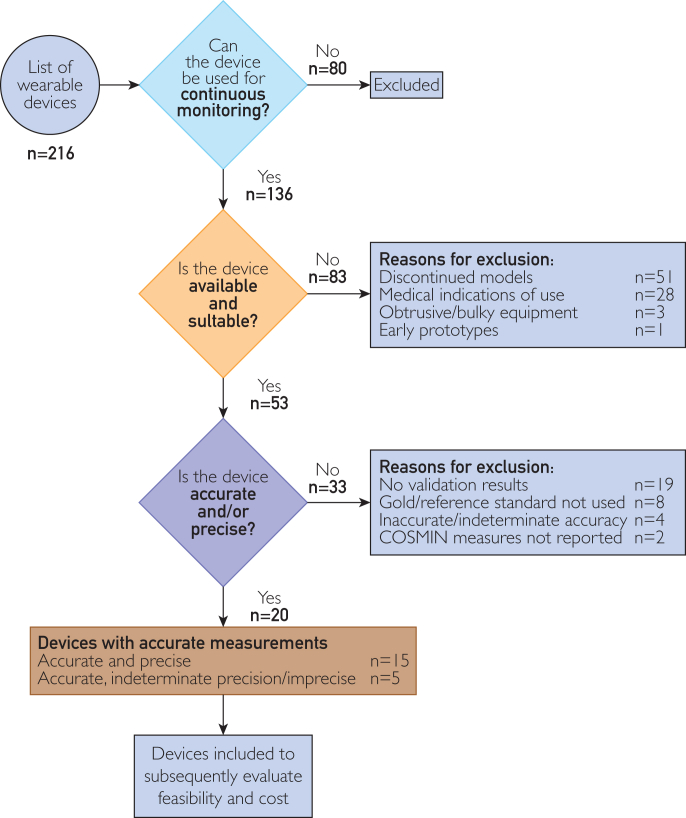
Selection of wearable devices for continuous monitoring of cardiovascular functions in relatively healthy community-dwelling adults. Beginning with a list of 216 devices, the devices were evaluated for their continuous monitoring capability, whether they were available and suitable, and whether they were accurate and/or precise. Finally, 20 valid devices remained for subsequent evaluation of their feasibility of use and costs entailed. COSMIN, COnsensus-based Standards for the selection of health Measurement INstruments.

### Criterion 3: Technical Performance (Accuracy and Precision)

The validation results for each measurement of the valid devices are given in Table 1. After excluding devices that were not validated with the gold/reference standard, did not have performance measures reported on the basis of the COSMIN tool criteria,^13^ or were inaccurate/have indeterminate accuracy (Appendix 1, available online at https://www.mcpdigitalhealth.org/), 20 devices remained for further evaluation.

### Criterion 4: Feasibility of Use

On the basis of the 9 parameters for feasibility of use (Table 2),^8^ the advantages and disadvantages of the 20 devices (Aktiia Bracelet, Apple Watch Series 9, Biobeat Wrist Monitor, Biofourmis Everion+, Cardiac Sense Wristwatch, Corsano Cardiowatch Bracelet, Firstbeat Bodyguard 3 Heart Rate Monitor, Firstbeat Textile ECG Strap, Fitbit Charge 6, Garmin vívosmart 5, Hexoskin Smart Shirt, LiveMetric LiveOne, Omron HEM-6140T “HeartGuide”, Oura Ring Gen3, Polar H10 Heart Rate Sensor, Polar Vantage M2 Watch, Samsung Galaxy Watch7, VitalConnect VitalPatch RTM, WHOOP 4.0 Band, and Zephyr BioPatch HP) with valid cardiovascular measurements are provided (Appendix 2, available online at https://www.mcpdigitalhealth.org/) and summarized (Table 3). The Corsano Cardiowatch had 3 validated measurements (BP, HR, and HRV), and 9 other devices had 2 validated measurements. All devices, excluding Corsano Cardiowatch and Omron HeartGuide, were validated in a healthy adult population. There were 15 devices that reported high acceptability (ie, low discomfort): 12 wrist-based, 2 chest patches and 1 ring. No devices reported high usability as they required manual syncing, infrequent/daily charging/cleaning, or another device for data readout. The Aktiia Bracelet and Polar H10 Heart Rate Monitor exhibited a battery life exceeding 7 days. All but 3 devices (Biofourmis Everion+, VitalConnect VitalPatch RTM, and Zephyr BioPatch HP) could be sanitized and reused between users. There were 13 devices that had a data/user management platform and a user-interactive application. Eight devices provided control over firmware and software updates. The Firstbeat Bodyguard 3 Heart Rate Monitor and Hexoskin Smart Shirt had data management platforms that permitted raw data export and access to processing algorithms.

**Table 3:** The Feasibility of Using Valid Continuous Monitoring Wearable Devices in Clinical Trials to Measure Cardiovascular Function in Relatively Healthy Community-Dwelling Adults and the Cost per Device

Wearable device	User experience					Usage in research				Cost per device (USD)
	Measurements validated	Population validated	Acceptability	Usability	Battery life	Device setup/maintenance	Data management	Firmware/software updates	Data access	
Aktiia Bracelet										265
Apple Watch Series 9										399
Biobeat Wrist Monitor										2040
Biofourmis Everion+										—
Cardiac Sense Wristwatch										890
Corsano Cardiowatch Bracelet										423
Firstbeat Bodyguard 3 Heart Rate Monitor										295
Firstbeat Textile ECG Strap										168
Fitbit Charge 6										160
Garmin vívosmart 5										150
Hexoskin Smart Shirt										598
LiveMetric LiveOne										40,000^a^
Omron HEM-6140T “HeartGuide”										500
Oura Ring Gen3										299
Polar H10 Heart Rate Sensor										100
Polar Vantage M2 Watch										300
Samsung Galaxy Watch6										300
VitalConnect VitalPatch RTM										199
WHOOP 4.0 Band										384
Zephyr BioPatch HP										—

Usage in research and/or clinical practice: , high feasibility; , medium feasibility; , low feasibility. —, no information was obtained.

ECG, electrocardiogram.

Cost per device (in USD) is the quoted price on the basis of a minimum order quantity of 50 devices for 1 year’s usage for comparison and is updated as of July 1, 2024.

^a^Minimum order of 40,000 USD required for devices, subscriptions/licenses, and software.

Although the feasibility parameters were not linearly additive, devices with more green (high feasibility) and fewer red (low feasibility) circles provided a proxy for selecting a device. Thus, the Corsano Cardiowatch Bracelet and Oura Ring Gen3 (both with 5 green and no red circles) emerged as potential choices. Considering a specific clinical scenario—continuous monitoring in community-dwelling adults aged 40 to 60 years undergoing a geroprotective intervention—the Oura Ring Gen3 may be most suitable. It can measure HR and HRV, had been validated in healthy adults, and has a small, nonobtrusive design (ring vs wrist-based) for low discomfort. Both devices can be sanitized and reused, has a data/user management platform and user-interactive app, and allow control over firmware and software updates and raw data exports.

### Criterion 5: Cost Evaluation

Costs are listed in Appendix 2 (available online at https://www.mcpdigitalhealth.org/). Devices with the lowest cost per device included the Polar H10 Heart Rate Sensor and Garmin vívosmart 5. Devices with the highest cost per device included the LiveMetric LiveOne and Biobeat Wrist Monitor. The Oura Ring (299 USD/unit) was cheaper than the Corsano Cardiowatch (423 USD/unit) and could be the more optimal choice in the clinical scenario previously described. Information on subscription and auxiliary costs are provided in Appendix 2 (available online at https://www.mcpdigitalhealth.org/).

## Discussion

Of 216 devices considered, 20 devices proved suitable for continuous monitoring in community-dwelling adults. Health wearables, such as the Apple Watch Series 9, Fitbit Charge 6, Garmin vívosmart 5, Oura Ring Gen3, Polar Vantage M2 Watch, Samsung Galaxy Watch7, and the WHOOP 4.0 Band, were included. As of July 1, 2024, 18 of these devices had been validated in a healthy adult population. Each device varied in feasibility of use and costs relating to the device, system licenses, and subscriptions.

Continuous and consistent monitoring is important during clinical studies for the regular collection of data with minimal interruptions or gaps, ensuring continuous and reliable insights. This is particularly crucial when studying physiologic changes, cyclic habits, and health trends, as gaps in data can reduce the accuracy and reliability of results. In combination, continuous and consistent monitoring over extended periods complement health status assessments conducted during intermittent clinic visits.^4^^,^^35^ The continuous and consistent collection of data is invaluable for clinical interventions aimed at understanding how certain lifestyle behaviors influence the aging process. Therefore, choosing an appropriate device is crucial for successful data collection on cardiovascular function in clinical interventional trials. The device must be accurate, consistently worn by users, easily integrated into the workflows of researchers and HCPs and cost-effective. Such a device can help identify indicators of aging that may be affected by the intervention. Privacy and data security are also critical considerations. Although the specific approaches to data security may vary depending on the research or clinical workflow, the practical guide addresses these concerns under the feasibility criteria. Various published reviews provide a more in-depth exploration of privacy and security issues,^36^^,^^37^ offering guide users further resources to tailor device selection to their needs.

The application of the guide aids in systematically filtering out devices unfit for continuous monitoring, ensuring availability and suitability, as well as obtaining accuracy and feasibility information about the device and costs of accompanying software and subscriptions. This information is then used to make an informed decision during device selection. The chosen device, however, depends on the unique research/clinical objectives and resource constraints, which can be considered as the feasibility parameters are evaluated. The guide suggests that the utility of a device increases with a higher number of valid measurements, enhancing data collection efficiency and reducing the burden on users.^8^ In this situation, the Corsano Cardiowatch Bracelet may be a suitable device because it can measure BP, HR, and HRV. Low discomfort is important for acceptability, making wrist-based and ring-like devices preferable in this situation, such as the Apple Watch Series 9, Fitbit Charge 6, Garmin vívosmart 5, Oura Ring Gen3, and WHOOP 4.0 Band. On the contrary populations from different generations, socioeconomic backgrounds, or with specific needs (eg, visual impairments or limited digital literacy) may require tailored approaches to device selection. For example, individuals with visual impairments may benefit from devices with longer battery life, minimal interaction requirements, and user-friendly features such as tactile buttons or larger text.^5^ Devices like the Aktiia Bracelet and the Polar H10 Sensor are examples that align with these criteria by requiring minimal interaction and offering features suitable for low-vision users. Similarly, individuals with limited digital literacy may face challenges with device setup, data syncing, or application-based feedback. For such populations, devices with simplified workflows, automated data syncing, or offline usability may be more appropriate. The practical guide’s feasibility parameters, such as acceptability and usability, allow users to assess and prioritize these features to meet the specific needs of their target population. By considering these factors early in the selection process, researchers, and HCPs can identify devices that enhance adherence and accessibility for diverse user groups. All the 20 selected devices reported continuous monitoring capability and were accurate. A few of those devices were designed for and, therefore, more suitable for sports monitoring, which may be useful in lifestyle and habit interventions regarding activity and exercise. Such devices include the Firstbeat Textile ECG Strap, Polar H10 Heart Rate Monitor, and Zephyr BioPatch HP because their data management systems were designed with data collection and analysis of short training sessions in mind. These devices also tend to be chest straps, which help to reduce motion artifacts and allow wider ranges of motion during exercise.^38^ Regarding data management and access to data, if there is no management software or the device user needs to manually share collected data, this may become inconvenient and cumbersome logistically. The following devices have a user-interactive application for data syncing but no data management platform, however, for the researcher/HCP: the Omron HeartGuide, Samsung Galaxy Watch7, and WHOOP 4.0 Band. Thus, these devices may not be as appropriate for use in clinical studies. The interoperability of wearable devices with commercial electronic databases is also a critical consideration in both research and clinical settings. Devices that are compatible with widely used clinical databases and software platforms can streamline data integration, reduce manual data processing, and improve the efficiency of workflows for HCPs and researchers.

This article is the first application of a practical guide^8^ for describing how to select a wearable device for clinical studies. Another strength is the step-by-step process illustrated to show how a list of over 200 potential devices could be narrowed down to 20 devices for consideration, which becomes more manageable to compare. The feasibility assessment also incorporated metrics for wearability, user comfort, and long-term adherence under the acceptability and usability parameters. These considerations are critical for ensuring that selected devices align with real-world conditions and can be worn consistently without disrupting daily activities. By addressing user burden and adherence, the framework emphasizes practicality and enhances the real-world applicability of continuous monitoring devices. One limitation, however, is that this selection focused only on devices measuring cardiovascular function. If collection of other measurements is desired, then additional evaluation is required. Another limitation of this study is the validation of device accuracy and precision relied on previously published literature and manufacturer-reported data rather than conducting independent testing. This approach allowed the evaluation of a broad range of devices without the need for significant investment in equipment and facilities, but it could have also introduced variability. Some validation studies adhered to established guidelines,^13^^,^^39^ ensuring reliable assessments of device performance. However, others lacked clear thresholds for accuracy and precision (ie, the studies did not report predefined thresholds) or focused only on 1 aspect of validation, leading to unequal comparisons of device accuracy and precision.^21^^,^^33^^,^^40^ This variability underscores the importance of transparent and standardized reporting in validation studies to support more consistent device evaluations. Using the COSMIN framework and international clinically accepted requirements helped provide guardrails to ensure that only studies meeting methodologic standards were included. Moving forward, the development of more accessible validation methodologies and resources could enable independent testing and improve the reliability of the device selection process. Furthermore, researchers and HCPs should remain aware of the inherent variability in existing validation data when applying the practical guide to their unique situations. It is difficult, also, to evaluate feasibility from an ethical and cultural standpoint, such as evaluations on data privacy, participant safety, and social/psychological comfort, which may vary in different cultures and populations and require increased input from experts on those topics during device selection.^7^ These context-specific concerns are important to examine when designing a study and determining which devices to use.

In conclusion, this application of the practical guide provides a concrete example to select wearable devices for continuous monitoring of cardiovascular functions in healthy community-dwelling adults participating in clinical studies. The step-by-step process outlined serves as a valuable reference for researchers, HCPs, and device users navigating the complexities of wearable device selection.

## Potential competing interests

The authors report no competing interests.
