# Supplementary material for: Selecting Wearable Devices to Measure Cardiovascular Functions in Community-Dwelling Adults: Application of a Practical Guide for Device Selection
Source: Mayo Clin Proc Digit Health. 2025 Mar 12;3(2):100202. doi: 10.1016/j.mcpdig.2025.100202 (PMC12190978; doi:10.1016/j.mcpdig.2025.100202)
Supplement: Supplementary material [file mmc1.pdf]

SUPPLEMENTARY MATERIAL

Selecting wearable devices to measure cardiovascular functions in community-dwelling adults: Application of a practical guide for device selection

Jessica K. Lu, Weilan Wang, Jorming Goh, Andrea B. Maier

Healthy Longevity Translational Research Programme, Yong Loo Lin School of Medicine, National University of Singapore, Singapore, Singapore (J K Lu MEng, W Wang PhD, J Goh PhD, Prof A B Maier MD);

Academy for Healthy Longevity, Yong Loo Lin School of Medicine, National University of Singapore, Singapore, Singapore (J K Lu, W Wang, Prof A B Maier);

Centre for Healthy Longevity, National University Health System, Singapore, Singapore (J K Lu, W Wang, J Goh, Prof A B Maier);

Department of Physiology, Yong Loo Lin School of Medicine, National University of Singapore, Singapore, Singapore (J Goh);

Department of Human Movement Sciences, @AgeAmsterdam, Faculty of Behavioural and Movement Sciences, Vrije Universiteit Amsterdam, Amsterdam Movement Sciences, Amsterdam, the Netherlands (Prof A B Maier)

Corresponding author:
Prof Dr Dr Andrea B Maier
Department of Human Movement Sciences, Faculty of Behavioural and Movement Sciences, Vrije Universiteit Amsterdam, Amsterdam Movement Sciences,
Van der Boechorstsraat 7, 1081 BT Amsterdam, the Netherlands
Email: **a.b.maier@vu.nl**

Table of Contents

Appendix 1. Selection of wearable devices for measuring cardiovascular function that can be used for continuous monitoring in community-dwelling adults and have valid measurements. **3**

Appendix 2. The advantages and disadvantages of the 20 valid wearable devices for measuring cardiovascular functions in community-dwelling adults based on the nine feasibility of use parameters and cost. **15**

Appendix 3. Device manufacturers contacted for additional information on feasibility and cost of valid wearable devices for continuous monitoring in community-dwelling adults. **20**

References **21**

Appendix 1. Selection of wearable devices for measuring cardiovascular function that can be used for continuous monitoring in community-dwelling adults and have valid measurements.

Remote monitoring device	Continuous monitoring	Reason for exclusion	Current model (if applicable)	Accuracy (measurement error)			Precision (reliability)			Reason for exclusion	Included
				At rest	LPA	MVPA	At rest	LPA	MVPA		
A&D TM-2441	✗										
A&D UA-651BLE BP Monitor	✗										
A&D UA-767PBT-Ci	✗										
A&D UA-772	✗										
Aktiia Bracelet	✓	—	—	Bias ± SEm[1]<br>SBP: 0.46 ± 7.75<br>DBP: 0.39 ± 6.86	—	—	PCC[1]<br>SBP: $r = 0.92$<br>DBP: $r = 0.83$	—	—	—	✓
AliveCor KardiaMobile®	✗										
AliveCor KardiaMobile® 6L	✗										
AliveCor KardiaBand	✗										
Amazfit ECG recorder	✓	Discontinued									
Amazfit Health Band 1S	✓	Outdated model	Amazfit Band 7	—	—	—	—	—	—	No validation†	
ANSWatch Model TS-0411	✗										
Apple iPhone 4S	✗										
Apple iPhone 6	✗										
Apple iPhone 6S	✗										
Apple iPhone 8	✗										
Apple Watch	✓	Discontinued									
Apple Watch 2 Nike+	✓	Discontinued									
Apple Watch Series 0	✓	Discontinued									
Apple Watch Series 1	✓	Discontinued									
Apple Watch Series 2	✓	Discontinued									
Apple Watch Series 3	✓	Discontinued									
Apple Watch Series 4	✓	Discontinued									

Remote monitoring device	Continuous monitoring	Reason for exclusion	Current model (if applicable)	Accuracy (measurement error)			Precision (reliability)			Reason for exclusion	Included
				At rest	LPA	MVPA	At rest	LPA	MVPA		
Apple Watch Series 5	✓	Discontinued									
Apple Watch Series 6	✓	Outdated model	Apple Watch Series 9	Bias (LoA)[2]<br>HR: –0.11 (–5.84, 5.63)<br>SpO_2: –0.23 (–3.49, 3.04)	—	—	PCC[2]<br>HR: $r = 0.98$<br>SpO_2: $r = 0.89$	—	—	—	✓
ASUS VivoWatch BP	✓	—	—	—	—	—	—	—	—	No validation†	
AutoSense chest band	✓	—	—	—	—	—	—	—	—	Reference standard not used[3]	
AViTA BPM65ZB	✗										
Basis B1	✓	Discontinued									
Beat-by-Beat BP Monitor	✓	—	—	—	—	—	—	—	—	No validation†	
Biobeat Wrist Monitor	✓	—	—	Bias ± LoA[4]<br>SBP: –0.08 (–7.06, 6.90)<br>DBP: 0.00 (–6.88, 6.87)	—	—	ICC[4]<br>SBP: 0.99<br>DBP: 0.98	—	—	—	✓
Biostrap Wristband	✓	—	—	—	—	—	—	—	—	No validation†	
BodiMetrics Performance Monitor	✗										
BodyTrace BP monitor	✗										
BIC model YK-80A	✗										
Biocom Heart Tracker	✗										
Biologix Oxistar	✓	Sleep apnea monitoring									
Biovotion Everion® bracelet	✓	Outdated model	Biofourmis Everion+™	Bias (LoA)[5]<br>HR: –1.06 (–9.59, 7.47)<br>HRV: –0.91 (–7.35, 5.53)	Bias (LoA)[5]<br>HR: –0.23 (–4.05, 3.58)<br>HRV: –5.74 (–12.31, 0.83)	Bias (LoA)[5]<br>HR: 0.29 (–11.73, 12.30)	ICC (CI)[5]<br>HR: 0.97 (0.97, 0.98)<br>HRV: 0.94 (0.90, 0.96)	ICC (CI)[5]<br>HR: 0.99 (0.99, 0.99)<br>HRV: 0.14 (–0.09, 0.48)	ICC (CI)[5]<br>HR: 0.95 (0.95, 0.95)	—	✓
BPLab device	✓	ABPM									
Cardiac Designs ECG Check device	✗										

Remote monitoring device	Continuous monitoring	Reason for exclusion	Current model (if applicable)	Accuracy (measurement error)			Precision (reliability)			Reason for exclusion	Included
				At rest	LPA	MVPA	At rest	LPA	MVPA		
CardiacSense Wristwatch	✓	—	—	Bias (LoA)[6]<br>RRi: –0.1 (–29.2, 29)	—	—	PCC[6]<br>RRi: r = 0.99	—	—	—	✓
CardioMem CM3000	✓	Holter ECG									
CardioPoint H100 Holter	✓	Holter ECG									
CardioQVARK® iPhone case	✗										
Cardioskin smart T-shirt	✓	Prototype[7]									
ChoicMMed OxyWatch	✗										
Cloud DX BP Monitor	✗										
Coala Heart Monitor	✗										
Contec Medical ABPM50	✓	ABPM									
Contec CMS50EW pulse oximeter	✗										
Contec SAT-300 finger pulse oximeter	✗										
Corsano Cardiowatch 287 Bracelet	✓	—	—	Bias (LoA)[8]<br>SBP: –0.17 (–8.74, 8.4)<br>DBP: 0.2 (–6.96, 7.37)<br>Bias (LoA)[9]<br>HR: –0.06 (–3.89, 3.77)<br>RRi: –1 (–173, 171)	—	—	PCC[8]<br>SBP: r = 0.985<br>DBP: r = 0.961<br>PCC (CI)[9]<br>HR: r = 0.99 (0.99, 0.99)<br>RRi: r = 0.89 (0.89 – 0.90)	—	—	—	✓
Desay Electronics Algoband F8	✓	—	—	—	—	—	—	—	—	No validation†	
ECG247 Smart Heart Sensor	✓	Heart arrhythmia detection									
ECIL Remote Monitoring device	✓	Obtrusive finger attachment									
EMAY EMO-80 Pulse Oximeter	✓	Overnight sleep monitoring									

Remote monitoring device	Continuous monitoring	Reason for exclusion	Current model (if applicable)	Accuracy (measurement error)			Precision (reliability)			Reason for exclusion	Included
				At rest	LPA	MVPA	At rest	LPA	MVPA		
Empatica E4 wristband	✓	Discontinued									
Everlast TR10 Watch	✓	Discontinued									
Firstbeat Bodyguard 2 HR Monitor	✓	Outdated model	Firstbeat Bodyguard 3	Bias[10]<br>RRi: –0.70<br>HRV (RMSSD): 7.99	Bias[10]<br>RRi: –0.30<br>HRV (RMSSD): 4.44	Bias[10]<br>RRi: 1.50<br>HRV (RMSSD): 20.54	—	—	—	Sufficient accuracy, indeterminate precision	✓
Firstbeat Textile ECG Strap	✓	—	—	Bias[11] (LoA)<br>HR: 0 (0, 0)<br>HRV: –7.37 (–33.00, 18.27)	—	—	CCC[11]<br>HRV (RMSSD): 0.76	—	—	—	✓
Fitbit 2	✓	Discontinued									
Fitbit Alta	✓	Discontinued									
Fitbit Alta HR	✓	Discontinued									
Fitbit Blaze	✓	Discontinued									
Fitbit Charge 2	✓	Discontinued									
Fitbit Charge 3	✓	Discontinued									
Fitbit Charge 4	✓	Outdated model	Fitbit Charge 6	Charge 2:<br>Bias (LoA) [12]<br>HR: –1.26 (–12.4, 9.90)<br>Charge 4:<br>Bias (LoA)[13]<br>HR: 3.36 (–18.98, 25.70)	Charge 4:<br>Bias (LoA)[13]<br>HR: –0.98 (–20.68, 18.73)	—	Charge 2:<br>CCC (CI) [12]<br>HR: 0.89 (0.84, 0.92)<br>Charge 4:<br>CCC[13]<br>HR: 0.203<br>PCC<br>HR: 0.257	Charge 4:<br>CCC[13]<br>HR: 0.348<br>PCC<br>HR: 0.408	—	Sufficiently accurate, but not precise	✓
Fitbit Charge HR	✓	Discontinued									
Fitbit Charge HR 2	✓	Discontinued									
Fitbit Charge HR 3	✓	Discontinued									
Fitbit Inspire HR	✓	Outdated model	Fitbit Inspire 3	—	—	—	—	—	—	Reference standard not used[14]	
Fitbit Ionic	✓	Discontinued									
Fitbit Surge	✓	Discontinued									
Fitbit versa	✓	Discontinued									
Fitbit versa 2	✓	Outdated model	Fitbit versa 4	—	—	—	—	—	—	Reference standard not used[15]	
ForaCare Diamond Cuff	✗										
Garmin Forerunner 945	✓	Outdated model	Garmin Forerunner 965	—	—	—	—	—	—	Reference standard not used[16]	

Remote monitoring device	Continuous monitoring	Reason for exclusion	Current model (if applicable)	Accuracy (measurement error)			Precision (reliability)			Reason for exclusion	Included
				At rest	LPA	MVPA	At rest	LPA	MVPA		
Garmin vívoactive 3	✓	Outdated model	Garmin vívoactive 5	—	—	—	—	—	—	No validation†	
Garmin vívofit HR	✓	Discontinued									
Garmin vívosmart	✓	Discontinued									
Garmin vívosmart 3	✓	Discontinued									
Garmin vívosmart 4	✓	Outdated model	Garmin vívosmart 5	Bias (CI)[17] HR: 1.76 (–19.98, 23.50)	Bias (CI)[17] HR: –16.80 (–67.58, 33.98)	Bias (CI)[17] HR: –43.44 (–123.83, 36.95)	ICC[17] HR: 0.76	ICC[17] HR: 0.34	ICC[17] HR: 0.11	— (Garmin vívosmart HR)	✓
Garmin vívosmart HR	✓	Discontinued									
Garmin vívosmart HR+	✓	Discontinued									
Garmin vívosport™	✓	—	—	—	—	—	—	—	—	No validation†	
Globalcare GCE603 BP Monitor	✗										
HealthSTATS Bpro	✓	—	—	Bias (LoA)[18] SBP: –0.87 (–28.5, 26.76) DBP: –4.49 (–27.03, 18.04)	—	—	—	—	—	Insufficient accuracy[18] and indeterminate precision	
Health&Life HL868ED BP Monitor	✗										
HeartMan wristband	✓	—	—	—	—	—	—	—	—	Reference standard not used[19]	
Hexoskin Smart Shirt	✓	—	—	CV ±SD (%)[20] HR: 0.52 ± 0.55	CV ±SD (%)[20] HR: 0.79 ± 0.77	—	ICC[20] HR: 0.99	ICC[20] HR: 0.99	—	—	✓
Huawei Watch 2	✓	Outdated model	Huawei Watch 3 (Pro)		—	—	—	—	—	No validation†	
Huawei Watch GT2 Pro ECG edition	✓	Outdated model	Huawei Watch GT 4	—	—	—	—	—	—	No validation†	
iHealth Air PulseOx3M	✗										
iHealth Neo BP device	✗										
iHealth Lab BP3L Monitor	✗										
ImageONE Duranta	✓	Holter ECG									
InBodyWATCH cuffless device	✓	—	—	—	—	—	—	—	—	No validation†	

Remote monitoring device	Continuous monitoring	Reason for exclusion	Current model (if applicable)	Accuracy (measurement error)			Precision (reliability)			Reason for exclusion	Included
				At rest	LPA	MVPA	At rest	LPA	MVPA		
Itamar Watch-PAT 200	✓	Sleep apnea monitoring									
iWRAP theranostic prototype	✓	Compression device									
Kenek Edge pulse oximeter probe	✗										
Konica Minolta PULSOX-Me300	✓	Overnight oximetry monitoring									
Lenovo ZUK Z2 X	✗										
Life Plus Dona Care Watch	✓	—	—	—	—	—	—	—	—	No validation†	
LiveMetric LiveOne	✓	—	—	Bias (LoA)[21]<br>SBP: 0.2 (–20.0, 20.4<br>DBP: 0.9 (–14.1, 15.9)		—	PCC[21]<br>SBP: $r = 0.91$<br>DBP: $r = 0.85$	—	—	—	✓
Masimo MightSat	✗										
m-Health Solutions Pocket-ECG	✓	Holter ECG									
Microlife BP A6 PC BP Monitor	✗										
Microlife BP B3 AFIB Monitor	✗										
Microlife WatchBP-Home A Monitor	✗										
Microlife WatchBP O3 AFIB Monitor	✓	ABPM									
Mobil-O-Graph BP Monitor	✓	ABPM									
Mobil-O-Graph Arteriograph	✓	ABPM									
Mobil-O-Graph NG	✓	ABPM									
Mobil-O-Graph PWA	✓	ABPM									
MotionSense HRV	✓	—	—	—	—	—	—	—	—	No validation†	
Movesense HR+ Sensor	✓	—	—	—	—	—	—	—	—	No validation†	
Nonin 3230 finger pulse oximeter	✗										
Nonin G92 finger pulse oximeter	✗										
Nonin Onyx II pulse oximeter	✗										

Remote monitoring device	Continuous monitoring	Reason for exclusion	Current model (if applicable)	Accuracy (measurement error)			Precision (reliability)			Reason for exclusion	Included
				At rest	LPA	MVPA	At rest	LPA	MVPA		
Nonin WristOx$_2$® Model 3150	✓	Obtrusive finger attachment									
Nonin WristOx$_2$® Model 3250	✓	Obtrusive finger attachment									
Nordic Brain Cerebri	✗										
Novacor Diasys 3 Plus	✓	ABPM									
Nuvo-Group Invu Belt	✓	For pregnancy care									
Omron Evolv® BPM	✗										
Omron HEM-6410T "HeartGuide"	✓	—	—	Bias ± SD[22]<br>SBP: 3.4 ± 16.4<br>DBP: –3.2 ± 11.1<br>PR: –0.1 ± 6.1	Bias ± SD[22]<br>SBP: 2.4 ± 18.8<br>DBP: –1.3 ± 10.2<br>PR: –2.1 ± 7.8	—	PCC[22]<br>SBP: r = 0.63<br>DBP: r = 0.64<br>PR: r = 0.87	PCC[22]<br>SBP: r = 0.64<br>DBP: r = 0.75<br>PR: r = 0.79	—	BP measurements are insufficiently precise, but sufficiently accurate. PR measurements are accurate and precise.	✓
Omron HEM-4030	✗										
Omron HEM-7080IC	✗										
Omron HEM-7080-ITZ	✗										
Omron HEM-7080-ITZ2	✗										
Omron HEM-7251G	✗										
Omron HEM-7252G-HP	✗										
Omron HEM-7320T	✗										
Omron HEM-7600T	✗										
Omron HEM-9200T	✗										
Omron M3 Intellisense	✗										
Omron M6 Intellisense	✗										
Omron BP761N 7 Series	✗										
Omron VR BP710N 3 Series	✗										
Oura Ring	✓	Discontinued									
Oura Ring Gen2	✓	Outdated model	Oura Ring Gen3	Bias (CI)[23]	—	—	PCC[23]<br>HR: 0.99	—	—	—	✓

Remote monitoring device	Continuous monitoring	Reason for exclusion	Current model (if applicable)	Accuracy (measurement error)			Precision (reliability)			Reason for exclusion	Included
				At rest	LPA	MVPA	At rest	LPA	MVPA		
				HR: –0.63 (–1.38, 0.11)<br>rMSSD: –1.2 (–8.8, 6.5)<br>Bias (LoA)[11]<br>rMSSD: –2.33 (–17.85, 13.19)			rMSSD: 0.98<br>CCC[11]<br>rMSSD: 0.91				
Partron Co. Neofit	✓	Discontinued									
Peloton HR Band	✓	—	—	—	—	—	—	—	—	No validation†	
Petr Telegin BPLab	✓	ABPM									
Philips Health Watch DL8791	✓	Discontinued									
Polar A360® Activity Tracker	✓	Discontinued									
Polar H7 HR Sensor	✓	Discontinued									
Polar H10 HR Sensor	✓	—	—	Bias (LoA)[11]<br>HR: –0.32 (–1.92, 1.29)<br>RMSSD: –6.98 (–34.74, 20.78)	—	—	CCC[11]<br>RMSSD: 0.77	—	—	—	✓
Polar Ignite Watch	✓	Outdated model	Polar Ignite 3	—	—	—	—	—	—	Reference standard not used[16]	
Polar M430 Watch	✓	—	—	—	—	—	—	—	—	No validation†	
Polar M600 Watch	✓	Discontinued									
Polar OH1 HR Sensor	✓	Outdated model	Polar Verity Sense	—	—	—	—	—	—	Reference standard not used[24]	
Polar RS800CX	✓	Discontinued									
Polar S810i™ pulse watch + Polar chest band	✓	Discontinued									
Polar V800 Watch	✓	Outdated model	Polar Vantage V3	—	—	—	—	—	—	No validation†	
Polar Vantage M Watch	✓	Outdated model	Polar Vantage M2	SEM[25]<br>HR: 8.71	SEM[25]<br>HR: 6.98	SEM[25]<br>HR: 8.84	ICC (CI)[25]<br>HR: 0.42 (0.27, 0.73)	ICC (CI)[25]<br>HR: 0.78 (0.53, 0.90)	ICC (CI)[25]<br>HR: 0.58 (0.14, 0.80)	Accurate, but not precise, Authors say not acceptable for clinical use[25]	✓
Preventice BodyGuardian® Heart Monitor	✓	—	—	—	—	—	—	—	—	COSMIN tool statistical methods not used[26]	
Pulsesense PS-500B pulsimeter	✓	Discontinued									

Remote monitoring device	Continuous monitoring	Reason for exclusion	Current model (if applicable)	Accuracy (measurement error)			Precision (reliability)			Reason for exclusion	Included
				At rest	LPA	MVPA	At rest	LPA	MVPA		
Psychorus wristband	✓	Discontinued									
QardioArm Smart BPM	✗										
Recovery Plus HR Band	✓	Discontinued									
Samsung Galaxy phone	✗										
Samsung Galaxy Note 9	✗										
Samsung Galaxy S7	✗										
Samsung Galaxy S8	✗										
Samsung Galaxy S9	✗										
Samsung Galaxy Watch	✓	Discontinued									
Samsung Galaxy Watch Active2	✓	Discontinued									
Samsung Galaxy Watch3 (SM-R850)	✓	Outdated model	Samsung Galaxy Watch7	Bias (LoA) [13]<br>HR: 4.41 (–40.5, 49.33)<br>Bias (LoA)[27]<br>SBP: 0.4 (–8.8, 9.2)<br>DBP: 1.1 (–7.9, 10.1)	Bias (LoA) [13]<br>HR: 2.86 (–16.8, 22.51)	—	CCC[13]<br>HR: 0.51<br>PCC[13]<br>HR: $r = 0.56$<br>SBP: $r = 0.97$ [27]<br>DBP: $r = 0.92$ [27]	CCC[13]<br>HR: 0.44<br>PCC[13]<br>HR: 0.52	—	Not sufficiently accurate or precise for HR measurements; sufficiently accurate and precise for BP measurements	✓
Samsung Gear Sport Watch	✓	Discontinued									
Samsung Gear S3 Watch	✓	Discontinued									
Santiago TM Kit, pulse oximeter	✗										
Savvy ECG sensor	✓	—	—	—	—	—	—	—	—	No validation†	
Seers mobiCARE-MC100 ECG patch	✗										
SensiumVitals™ ECG Patch	✗										
Sensogram Sensoscan	✗										
Shanghai Berry Pulse Oximeter	✗										
Shenzhen Creative PC-68B Wrist Oximeter	✗										
Shimmer3 Mobile ECG (mECG)	✓	—	—	—	—	—	—	—	—	COSMIN tool statistical methods not used[28]	

Remote monitoring device	Continuous monitoring	Reason for exclusion	Current model (if applicable)	Accuracy (measurement error) At rest	LPA	MVPA	Precision (reliability) At rest	LPA	MVPA	Reason for exclusion	Included
Spacelabs BP Monitor	✓	ABPM									
Spacelabs 90207 BP Monitor	✓	ABPM									
Spacelabs 90217 BP Monitor	✓	ABPM									
Spacelabs Ultralite 90217	✓	ABPM									
Spacelabs Lifecard CF	✓	Holter ECG									
Spyder ECG monitor	✓	Holter ECG									
SunTech OSCAR-2 BPM	✓	ABPM									
TaiDoc Upright TD-3128 BPM	✗										
TaiDoc FORA P20b	✗										
Thought Technology Triple-Physiology Sensor	✗										
Upmood Band	✓	—	—	—	—	—	—	—	—	No validation†	
Venus Congestion Meter (VenCoM)	✗										
VitalConnect VitalPatch® RTM	✓	—	—	Bias (LoA)[29]<br>HR: 0.31 (–3.8, 4.42)	—	—	PCC[29]<br>HR: $r = 0.99$	—	—	—	✓
VitalSignum Beat2Phone	✓	Discontinued									
Wavelet Health wristband	✓	Discontinued									
Wellysis S-Patch Cardio Solution	✗										
WHOOP Strap 2.0	✓	Outdated model	WHOOP 4.0 Band	Bias (LoA)[30]<br>HR: –0.15 (–2.15, 1.85)<br>HRV: 4.8 (–7.8, 17.4)	—	—	—	—	—	Sufficient accuracy, indeterminate precision	✓
Withings BP Monitor	✗										
Withings Move ECG Watch	✗										
Withings Steel HR	✓	—	—	—	—	—	—	—	—	No validation†	
Xiaomi Mi Band Pulse 1S	✓	Discontinued									
Xiaomi Mi Band 2	✓	Discontinued									

Remote monitoring device	Continuous monitoring	Reason for exclusion	Current model (if applicable)	Accuracy (measurement error) At rest	Accuracy LPA	Accuracy MVPA	Precision (reliability) At rest	Precision LPA	Precision MVPA	Reason for exclusion	Included
Xiaomi Mi Band 3	✅	Discontinued									
Xiaomi Mi Band 4	✅	Discontinued									
Xiaomi Mi Band 5	✅	Outdated model	Xiaomi Smart Band 8	Average for all intensities[31]: Bias (CI) HR: 1.64 (–24.21, 27.48)	—	—	CCC (CI)[31] HR: 0.68 (0.54, 0.77)	CCC (CI)[31] HR: 0.80 (0.70, 0.86)	CCC (CI)[31] HR: 0.66 (0.52, 0.77)	Insufficiently accurate and precise[31]	
Zephyr BioHarness-3™	✅	Discontinued									
Zephyr BioPatch™ HP	✅	—	—	Bias (CI)[32] HR: –0.01 (–0.12, 0.10)	Bias (CI)[32] HR: 0.03 (–0.09, 0.14)	—	PCC[32] HR: $r = 0.99$	PCC[32] HR: $r = 0.99$	—	—	✅
Zephyr HxM HR Monitor	✅	—	—	—	—	—	PCC[33] HR: 0.78 RRi: 0.77	—	—	Indeterminate accuracy[33]	
ZOLL LifeVest™ WCD	✅	Wearable cardioverter									
Zewa UAM-900T BPM	❌										
Zewa UAM-910BT BPM	❌										
Al-Naami prototype	✅	—	—	—	—	—	—	—	—	Reference standard not used[34]	
Kaile prototype	❌										
Lan prototype	❌										
Mena prototype	✅	—	—	Bias ± SD[33] SBP: –7.77 ± 8.58 DBP: –1.02 ± 4.21	—	—	PCC[33] SBP: $r = 0.91$ DBP: $r = 0.97$ SCC[33] SBP: $\rho = 0.94$ DBP: $\rho = 0.98$	—	—	Only DBP met accepted BHS thresholds[35]	
Wan Bio Pad prototype	❌										
Yamakoshi prototype	❌										
Zhang prototype	❌										

For data that were not applicable or not reported, the notation "—" is used. Reference standard refers to the gold standard or clinical standard of measurement.
Unless otherwise stated, BP is in units of mmHg, HR in bpm, HRV (rMMSD) in ms, RRi in ms, and SpO_2 in %.
† No validation results in the articles from the systematic review, cited references, articles from a separate PubMed search, or from the brand website as of July 1, 2024.

Abbreviations: BHS, British Hypertension Society; bpm, beats per minute; CI, 95% confidence interval; DBP, diastolic blood pressure; mmHg, millimetres of mercury; HR, heart rate; HRV, heart rate variability; LoA, 95% limits of agreement; LPA, measurements taken at low intensity physical activity; PCC, Pearson's correlation coefficient, r; ms, millisecond; MVPA, measurements taken at moderate to high intensity physical activity; rMSSD, root mean square of successive differences; RRi, R-R interval; SBP, systolic blood pressure; SEm, standard error of measurement; SCC, Spearman's correlation coefficient, ρ; %, percentage.

Appendix 2. The advantages and disadvantages of the 20 valid wearable devices for measuring cardiovascular functions in community-dwelling adults based on the nine feasibility of use parameters and cost.

Wearable device (description, data collected)	Advantages	Disadvantages
**Aktiia Bracelet**[36]<br><br>Medical device approved for continuous monitoring at home<br><br>Average of all BP readings taken within a two-hour time window	1) BP<br>2) Hypotensive/hypertensive outpatients, ages 21–65 years (includes healthy adults)[1]<br>3) High: wristband-like, low discomfort<br>4) Manual syncing to app; infrequent charging<br>5) Nine days<br>6) Reusable, not water-resistant<br>7) Each measurement saved in bracelet, then transmitted to mobile app during syncing; data shown on web dashboard<br>8) Automatic updates for Aktiia App (toggled On/Off)<br>9) Web dashboard receives de-identified user data in real-time; raw data exports available<br>10) Mobile app is free	1) Measurements not validated: HR<br>4) Measurements taken only during resting periods; requires calibration once every 30 days<br>10) 265 USD/device; orders accepted only from Switzerland, Germany, France, Austria, Italy, UK, and Republic of Ireland
**Apple Watch Series 9**[37]<br><br>Consumer smartwatch<br><br>Average of HR, HRV, SpO_2, and temperature readings of various durations, activity time and intensity, and sleep staging	1) HR, SpO_2<br>2) Healthy adults older than 18 years[2]<br>3) High: wristband-like, low discomfort<br>4) Automatic measurements and data syncing to Health app in real-time<br>6) Reusable, water resistant (up to 50 m)<br>7) HealthKit-enabled Health app (iOS and WatchOS); data/user management platform<br>8) Automatic updates for device (toggled On/Off)<br>9) Third-party apps and web platform can request permission to retrieve user data	1) Measurements not validated: HRV<br>4) Frequent charging (every one to two days)<br>5) 18 hours<br>7) Custom app development required if more than native app data collection is desired<br>10) 446 USD/device
**Biobeat Wrist Monitor**[38]<br><br>Remote monitoring solutions for hospital at home and clinical trials and research<br><br>Average of vital signs, including PP, BP, SpO_2, PR, RR, SVR, ECG, HRV, MAP, temperature, SV, CO, and CI	1) BP<br>2) Healthy, some individuals diagnosed with hypertension, diabetes, and high cholesterol, ages 8–100 years[4]<br>3) High: wristband-like, low discomfort<br>4) Automatic syncing to cloud via Biobeat app, infrequent charging (every five days)<br>7) Cloud-based monitoring platform with smart alert system, department dashboard, and historical data on users; real-time data available from user syncing measurements with mobile app<br>9) Reports exported from monitoring platform	1) Measurements not validated: HR, HRV, SpO_2<br>5) Five days<br>6) Measurements pre-set but alert trigger thresholds customizable for each user; sampling frequency is adjustable; resuable, not waterproof*<br>8) Firmware version does not change; software updates cannot be aborted to certain clients*<br>10) Cost: 2040 USD/unit*
**Biofourmis Everion+™** [39]<br><br>Remote monitoring solution for medical care delivery and clinical trials<br><br>Collects vital signs data including HR, SpO_2, and RR	1) HR, HRV<br>2) Healthy adults ages 23–54 years[5]<br>3) Medium: armband-like, medium discomfort<br>7) User app for interactions with researcher/HCP, web monitoring platform for researcher/HCP<br>9) Data exports on web monitoring platform	1) Measurements not validated: SpO_2<br>4) Uploading passive/manual: NA<br>5) Battery life: NA<br>6) Device setup/maintenance: NA<br>8) Firmware/software update: NA<br>10) Cost: NA
**Cardiac Sense Wristwatch**[40]<br><br>Wearable medical heart rate monitor	1) HR<br>2) Ambulatory, healthy adults (41.25 ± 18.63 years)[6]<br>3) High: wristband-like, low discomfort	1) Measurements not validated: SpO_2<br>5) 40 hours, or 1.67 days*

Wearable device (description, data collected)	Advantages	Disadvantages
Continuous monitoring of heart conditions and vital signs, especially detection of heart arrhythmias	4) Data syncing automatically while user app open on the forefront/in the background*<br>6) IP-67 water resistant, can be sanitized with medical wipes that include alcohol; device intended for long-term and continuous monitoring, therefore, intended for personal use and not recommended for different users*<br>7) Mobile upload of data from user app to researcher/HCP web-based platform*<br>9) Raw data stored on Amazon Web Services servers in Germany, accessible by researcher/HCP through web-based platform*	6) Variables measured are not adjustable (subjected to regulatory approvals in each region); sampling frequency set by manufacturer (256 Hz for spot-checks, 64 Hz for passive routine monitoring)*<br>8) Firmware/software updates automatically pushed to all users worldwide, however, happens seldom*<br>10) Device cost: 890 USD[41]; User app is free. However, device only available in countries where product is registered with local health authorities.*
**Corsano Cardiowatch Bracelet**[42]<br><br>Wireless remote monitoring system for continuous collection of physiological data in home and healthcare settings<br><br>Collects activity and heart rhythm data (in particular, heart arrhythmia) by minute, second, or 25 Hz, 32 Hz, and 128 Hz	1) BP, HR, HRV (RRi)<br>3) High: wristband-like, low discomfort<br>4) Removal for hygiene purposes, charged for a few hours once every six days<br>5) 6–10 days*<br>6) Pair/sync with third-party apps via BLE API and SDK<br>7) Set up vital parameters and sampling frequency in cloud platform; water-resistant*<br>8) User prompted to update device firmware and mobile app[43]<br>9) Cloud platform for viewing real-time data, API/SDK solutions allow raw data exports	1) Measurements not validated: SpO_2<br>2) Population: cardiac patients (67.1 ± 11.1 years[8] and 60 ± 15 years[9])<br>10) Device cost: 423 USD*; Data management platforms:*<br>• Cloud & Portal: 105 USD/month (for 1–10 devices) or 527 USD/month (for 11–100 devices) with MOE of 6 months<br>• REST API licence: 4224 USD/year<br>• SDK licence: 19006 USD one-time + 10560 USD/year
**Firstbeat Bodyguard 3 Heart Rate Monitor**[44]<br><br>Standalone wearable data logger used with standard clinical electrodes<br><br>Suitable for accurate, unobtrusive, and long-term heart rate, ECG, and motion recording	1) HR, HRV (RMSSD)<br>2) Healthy adults (31.3 ± 10.7 years)[10]<br>3) High: chest patch, low discomfort<br>6) Reusable, not waterproof<br>9) Researcher/HCP has access to raw data and processing algorithms (actual data analysis conducted in another program, e.g., Kubios)	4) External device required for data readout and upload to cloud platform<br>5) Battery life: 5 days; re-charge in between every measurement recommended<br>7) USB connectivity between device and software (installed onto local computer)<br>8) Firmware/software update: no answer*<br>10) Software price is included in the device price:*<br>• 316 USD/unit (1–9 units)<br>• 295 USD/unit (19–99 units)<br>• 263 USD/unit (100+ unit)<br>• Electrodes: 35 USD/box (one box includes 10 pouches containing 300 electrodes)
**Firstbeat Textile ECG Strap**[44]<br><br>Real-time monitoring, recovery monitoring, and performance management solution for team sports<br><br>Captures data on stress, recovery, and sports performance parameters such as HR recovery, max HR, and VO_2 max	1) HR, HRV (RRi)<br>2) For exercise, training, and sports coaching monitoring (athletes) (20.00 ± 1.38 years)[11]<br>3) Medium: chest strap, medium discomfort<br>6) Reusable, water-resistant *<br>7) Mobile app for the device user (Android/iOS) and for the coaches (iOS)*<br>9) Raw data collected can be exported into other platforms (via API)	4) Designed for during practice, in the gym, in games, during remote training; Requires an external readout device for data syncing<br>5) 160 hours, or 6.67 days (self-replaceable CR2025 batteries)*<br>8) Firmware/software update: no answer*<br>10) Device cost: 168 USD/unit (includes two straps); Software: 153 USD/device user/year*
**Fitbit Charge 6**[45]<br><br>Fitness tracker for monitoring changes in wellness using health metrics<br><br>Tracks trends in HR, HRV, SpO_2, breathing rate, sleep, activity, and more	1) HR<br>2) Healthy adults (24.2 ± 4.6 years)[13]<br>3) High: wristband-like, low discomfort<br>6) Reusable, water-resistant up to 50 metres<br>7) Fitbit mobile app linked to a Google account; researcher/HCP cloud server<br>8) Device-related updates announced, researcher/ HCP controls whether update is implemented	1) Measurements not validated: HRV, SpO_2<br>4) Automatic data syncing when Fitbit app is opened, infrequent charging<br>5) Up to seven days<br>10) 160 USD/device

Wearable device<br>(description, data collected)	Advantages	Disadvantages
	9) Daily summary data of study-registered user can be exported to Excel or CSV file; public web API for accessing data from other Fitbit devices and software developer's kit available[46]	
**Garmin vívosmart 5**[47]<br><br>Consumer fitness tracker for everyday monitoring of health<br><br>Tracks steps, heart rate, and energy levels	1) HR<br>2) Healthy adults (27.5 ± 6.0 years)[48]<br>3) High: wrist-based, low discomfort<br>4) Device charging approximately once per week; manual verification of data syncing to app<br>6) Pairs with Garmin Connect™ Mobile app for data syncing to Fitrockr data management platform; researcher/HCP can provide feedback[49]<br>7) Data collected and sampling frequency can be set;* Water-resistant (can swim with the device)<br>8) Researcher/HCP/device user notified of firmware/software updates for installation<br>9) Garmin Health connected ecosystem and SDK provides raw data access to researcher/HCP	1) Measurements not validated: SpO_2<br>5) Up to seven days<br>10) Costs: 150 USD/device; Fitrockr data management platform: 526 USD/month for 50 months (i.e., 6318 USD/year) (cheaper device licence costs with more licenses purchased)*
**Hexoskin Smart Shirt**[50]<br><br>Comfortable non-invasive smart clothing with textile sensors<br><br>Continuous monitoring of cardiorespiratory function, activity, and sleep	1) HR<br>2) Healthy adults (26.3 ± 5.9 years)[20]<br>3) Medium: skin-tight shirt worn underneath other clothing, medium discomfort<br>6) Machine washable (piling and stretching resistant) for reuse<br>9) Online dashboards (OneView*) to visualize, manage, and interpret data; Open Data API allows raw data exports for analysis in other software	1) Measurements not validated: HRV<br>4) Real-time data stored locally on SD card in different device located inside the shirt is transmitted to mobile app and stored on cloud server<br>5) Up to 36 hours, or 1.5 days<br>7) Up to 100+ days of recording; cloud-based platform to visualize data<br>8) Automatic, if need be, but data collection not affected*<br>10) 598 USD per shirt and smart device to record physiological biometric data; OneView license (unlimited seats and users): 1000 USD/year*
**LiveMetric LiveOne**[51]<br><br>Cuff-free continuous blood pressure and heart rate monitor<br><br>Real-time BP and HR measurements for people with hypertension and cardiovascular diseases	1) BP<br>2) Healthy adults (60.7 ± 15.2 years)[21]<br>3) High: wristband-like, low discomfort<br>4) Data syncing passively or actively synced*<br>5) 7 days<br>9) Data export in most formats (e.g., Excel, CSV, etc.)*	1) Measurements not validated: HRV<br>6) Three pre-sets of device sampling frequencies available; IP22 (not water resistant) but can be sanitized*<br>7) User app and data management cloud platform provided by manufacturer<br>8) Automatic firmware/software update for the user app, but researcher/HCP notified of updates for data management platform*<br>10) Currently not for individual sale, only offered to hospitals, RPM providers, etc.; minimum order 40000 USD for devices, subscriptions/licenses, and software*
**Omron HEM-6410T "HeartGuide"** [52]<br><br>Wearable digital blood pressure monitor<br><br>Measures BP and HR and detects arrythmias	1) BP, PR<br>2) ABPM outpatients ages 24–87 years[22]<br>3) High: wristband-like, low discomfort<br>6) Reusable, not water-resistant<br>7) OMRON Connect mobile companion app	4) Daily charging required<br>5) Approximately 2 days<br>8) Firmware/software update: NA<br>9) Data access: NA<br>10) 500 USD/device
**Oura Ring Gen3**[53]<br><br>Smart ring for fitness, stress, sleep, and health	1) HR, HRV (rMSSD)<br>2) Healthy adults (20.00 ± 1.38 years)[11]<br>3) High: ring-type, low discomfort<br>6) Reusable, water-resistant (up to 100 metres)<br>7) Oura mobile app for users, Oura Teams cloud platform for researcher/HCP	1) Measurements not validated: SpO_2<br>4) Manually ensure data is synced, charge at least once within seven days<br>5) Up to 7 days

Wearable device (description, data collected)	Advantages	Disadvantages
Optical heart rate sensor detects HR, HRV, and SpO_2	8) Researcher/HCP can control installation of firmware and cloud platform software updates*<br>9) Data access via Oura Teams and Oura API allows data integration to other platforms*	10) 299 USD/device; User membership: 7 USD/member/month; Oura Teams software: 1000 USD/year; Oura Teams seat: 10 USD/seat/month*
**Polar H10 Heart Rate Sensor**[54]<br><br>Electrical measurement-based heart rate chest strap monitor	1) HR, HRV (RMSSD)<br>2) Healthy adults (20.00 ± 1.38 years)[11]<br>3) Medium: chest strap, medium discomfort<br>5) 400 hours, or 16 days, (changeable coin battery)<br>6) Water resistant (can be used in water activities)<br>7) Polar Beat or Polar Flow mobile apps for user, Polar Flow web service for researcher/HCP<br>8) Control to update firmware via Polar Beat or Polar Flow web service<br>9) Researcher/HCP can access data on Polar Flow	1) Measurements not validated: SpO_2<br>4) Polar Pro chest strap washed daily; receiving device to view the data output must be kept in front of body and nearby for sufficient transmission range<br>6) Internal memory can store HR data of one training session (transfer to Polar Beat app after training)<br>10) 100 USD/device, mobile apps and web service are free
**Polar Vantage M2 Watch**[55]<br><br>Multisport watch with wrist-based heart rate tracking<br><br>Continuous heart rate feature measures HR 24/7	1) HR<br>2) Healthy adults (26.1 ± 3.4 years)[25]<br>3) High: wristband-like, low discomfort<br>6) Water resistant up to 30 m (suitable for swimming)<br>7) Polar Beat or Polar Flow mobile apps for user, Polar Flow web service for researcher/HCP<br>8) Notification to update Polar Flow mobile app or FlowSync on the computer<br>9) Data access using Polar Flow for detailed analysis of training results, activity, and sleep	4) Manual syncing to mobile app, infrequent charging<br>5) Up to 7 days in watch mode with continuous HR tracking<br>6) Requires pair and syncing to phone, setting up device parameters (time, date, etc.)<br>10) 300 USD/device, mobile apps and web service are free
**Samsung Galaxy Watch6**[56]<br><br>Consumer smartwatch with lifestyle tracking functions<br><br>Electrical heart sensor (ECG) and optical heart rate sensors allows for HR (and arrhythmia) and BP measurements	1) BP, HR<br>2) Healthy adults (24.2 ± 4.6 years) (HR)[13] and patients with Parkinson's disease (BP)[27]<br>3) High: wristband-like, low discomfort<br>6) Water-resistant up to 50 meters (no diving or high-pressure water activities)<br>7) Samsung Health and Samsung Health Monitor mobile apps for users	1) Measurements not validated: SpO_2<br>4) Daily charging required<br>5) Up to 40 hours, or 1.67 days<br>6) Accurate BP readings require oscillometric BP monitor calibration every 4 weeks<br>8) Firmware/software update: no answer*<br>9) Data access: no answer*<br>10) 300 USD/device
**VitalConnect VitalPatch® RTM**[57]<br><br>A battery-operated disposable, wearable monitor discretely worn on the chest (a mobile cardiac telemetry solution)<br><br>Direct measurement: ECG; derived measurement: HR and RRi, which can be processed into HRV metrics offline. Designed for arrhythmia detection.	1) HR<br>2) Healthy adults (32.5 ± 10.5 years)[29]<br>3) High: chest patch, low discomfort<br>4) User just needs to apply patch and leave it on during data collection period<br>6) IPX7 water-resistant for showers and daily wear<br>7) Mobile app for user, cloud-based management platform for researcher/HCP<br>9) Monitor user data collected on cloud-based user management platform	2) Measurements not validated: HRV, SpO_2<br>4) Third-party devices connect to the VistaTablet or VistaPhone (relay devices) via Bluetooth<br>5) 168 hours, or 7 days, after which it will cease to function (only used once)<br>6) Any data on biosensor must be transmitted before it turns off or data will be lost<br>7) Once biosensor is turned on and activated, it cannot be turned off; when disconnected from the relay device, data will be collected on biosensor for ~10 hours<br>8) Firmware/software update: NA<br>10) 199 USD/device
**WHOOP 4.0 Band**[58]<br><br>A wearable device without a screen for 24/7 biometric tracking	1) HR, HRV<br>2) Healthy adults (23.8 ± 5 years)[30]<br>3) High: wrist-based, low discomfort<br>6) IP-68 dust proof and water-resistant up to 10 metres for 2 hours	1) Measurements not validated: SpO_2<br>4) Data syncing to WHOOP mobile app; battery pack changed at least once within 7 days<br>5) Up to 4–5 days

Wearable device (description, data collected)	Advantages	Disadvantages
Monitors key vital signs, such as HR, SpO_2, skin temperature, respiratory rate, activity, and sleep	9) Health records and data trends can be exported into PDF format for each user via the user account side*	6) WHOOP mobile app for users; users can export their data for dissemination*<br>8) Users notified to install firmware updates in WHOOP app*<br>10) Membership: 32 USD/month (includes device and WHOOP app account)*
**Zephyr BioPatch™ HP**[59]<br><br>Physiological and biomechanical monitoring system designed for sports teams, elite athletes, first responders, and the military<br><br>ECG sensors measure HR, HRV, and SpO_2	1) HR<br>2) Healthy adults (21 ± 1 years)[32]<br>3) Medium: chest-strap or inserted into a t-shirt, medium discomfort<br>8) All firmware updates made available by support team, and researcher/HCP can choose to keep the same firmware version*<br>9) Raw data can be exported from management software	1) Measurements not validated: HRV, SpO_2<br>2) For sports performance management<br>4) Requires hand washing garments/soft or delicate wash cycles<br>5) Up to 35 hours, or 1.46 days<br>6) OmniSense data management software used for data visualization after exercise*<br>7) 36 different parameters for measurement; processed data sampling frequency: 1 Hz*<br>10) Cost: no answer*

Note: Details and currencies in USD are updated as of July 1, 2024.
The number-parameter pairings are as follows: 1) Measurements validated, 2) Population validated, 3) Acceptability, 4) Usability, 5) Battery life, 6) Device setup/maintenance, 7) Data management, 8) Firmware/software updates, 9) Data access, 10) Costs.
* Indicates the manufacturer was contacted via email or webform for this information.
NA indicates the information was not available, "no response" was defined as no reply after two follow-ups, and "no answer" was defined as the question was asked at least twice but the representative did not provide answers.
Abbreviations: ABPM, ambulatory blood pressure monitoring; API, application programming interface; BLE, Bluetooth Low Energy; BP, blood pressure; ECG, electrocardiogram/electrocardiograph; EUR, euros; GBP, British pound sterling; HR, heart rate; HRV, heart rate variability; IPX7, Ingress Protection X7 level of water resistance; NA, not available; RRi, R-R interval; RPM, remote patient monitoring; RMSSD, root mean square of successive differences; SDK, software development kit; SpO_2, blood oxygenation; UK, United Kingdom; USB, Universal Serial Bus; USD, United States dollars.

Appendix 3. Device manufacturers contacted for additional information on feasibility and cost of valid wearable devices for continuous monitoring in community-dwelling adults.

Manufacturer	First contact method	Response	Response from	Number of follow-ups if no response
Bio-beat	Email (October 6)	Three days after first follow-up email	Associate Business Development Manager	1st: Email (October 16)
Biofourmis	Webform (October 6)	NA	NA	1st: Webform (October 16)<br>2nd: Email (October 23)
Cardiac Sense	Email (October 6)	One day after first follow-up email	Sales Manager	1st: Email (October 16)
Corsano Health B.V.	Email (October 6)	One day after first email	Clinical Assistant	—
Firstbeat Technologies UK Ltd	Email (October 6)	One day after first email	Partner Executive	—
Garmin Health	Webform (October 6)	Three days after first webform	Community Manager	—
Hexoskin	Webform (October 6)	One day after first webform	Customer Support Specialist	—
LiveMetric	Email (October 10)	One day after third follow up (email on October 19)	CTO	1st: LinkedIn (October 11)<br>2nd: LinkedIn (October 16)<br>3rd: Email (October 19)
Omron	Webform (October 11)	NA	NA	1st: Webform (October 16)<br>2nd: Webform (October 23)
Oura	Webform (prior communications, September 2, 2022)	One day after first webform	Head of Health & Wellness Business Development	—
Samsung Electronics	Webform (October 11)	One day after first webform	Sales Development Representative	—
VitalConnect	Email (October 6)	NA	NA	1st: Email (October 16)<br>2nd: Email (October 23)
WHOOP	Webform (October 6)	One day after first webform	Membership Services Representative	—
Zephyr (Medtronic)	Email (October 11)	One day after first email	User Support Analyst	—

Details are updated as of December 31, 2023.
"—" indicates not applicable. Abbreviation: NA, not available.

References

1. Sola J, Vybornova A, Fallet S, Polychronopoulou E, Wurzner-Ghajarzadeh A, Wuerzner G. Validation of the optical Aktiia bracelet in different body positions for the persistent monitoring of blood pressure. *Sci Rep*. 2021;11(1):20644. doi:10.1038/s41598-021-99294-w
2. Spaccarotella C, Polimeni A, Mancuso C, Pelaia G, Esposito G, Indolfi C. Assessment of Non-Invasive Measurements of Oxygen Saturation and Heart Rate with an Apple Smartwatch: Comparison with a Standard Pulse Oximeter. *J Clin Med*. 2022;11(6):1467. doi:10.3390/jcm11061467
3. Ertin E, Stohs N, Kumar S, Raij A, al'Absi M, Shah S. AutoSense: unobtrusively wearable sensor suite for inferring the onset, causality, and consequences of stress in the field. presented at: Proceedings of the 9th ACM Conference on Embedded Networked Sensor Systems; 2011; Seattle, Washington. https://doi.org/10.1145/2070942.2070970
4. Nachman D, Gepner Y, Goldstein N, et al. Comparing blood pressure measurements between a photoplethysmography-based and a standard cuff-based manometry device. *Sci Rep*. Sep 30 2020;10(1):16116. doi:10.1038/s41598-020-73172-3
5. Barrios L, Oldrati P, Santini S, Lutterotti A. Evaluating the accuracy of heart rate sensors based on photoplethysmography for in-the-wild analysis. presented at: Proceedings of the 13th EAI International Conference on Pervasive Computing Technologies for Healthcare; 2019; Trento, Italy. https://doi.org/10.1145/3329189.3329215
6. Hochstadt A, Havakuk O, Chorin E, et al. Continuous heart rhythm monitoring using mobile photoplethysmography in ambulatory patients. *J Electrocardiol*. 2020;60:138-141. doi:10.1016/j.jelectrocard.2020.04.017
7. Fouassier D, Roy X, Blanchard A, Hulot JS. Assessment of signal quality measured with a smart 12-lead ECG acquisition T-shirt. *Ann Noninvasive Electrocardiol*. 2020;25(1):e12682. doi:10.1111/anec.12682
8. Ronner E, van Vliet M, Monnink SHJ, Kuiper MJ, Hoftijzer D, Constandse JC. Validation of a novel cuffless photoplethysmography-based wristband for measuring blood pressure according to the regulatory standard. 2023:
9. Blok S, Piek MA, Tulevski, II, Somsen GA, Winter MM. The accuracy of heartbeat detection using photoplethysmography technology in cardiac patients. *J Electrocardiol*. 2021;67:148-157. doi:10.1016/j.jelectrocard.2021.06.009
10. Parak J, Korhonen I. Accuracy of Firstbeat Bodyguard 2 beat-to-beat heart rate monitor. Tampere University of Technology; 2015. Accessed accessed June 1, 2023. https://www.firstbeat.com/wp-content/uploads/2015/10/white_paper_bodyguard2_final.pdf
11. Stone JD, Ulman HK, Tran K, et al. Assessing the Accuracy of Popular Commercial Technologies That Measure Resting Heart Rate and Heart Rate Variability. *Front Sports Act Living*. 2021;3:585870. doi:10.3389/fspor.2021.585870
12. Baek S, Ha Y, Park HW. Accuracy of Wearable Devices for Measuring Heart Rate During Conventional and Nordic Walking. *PM R*. 2021;13(4):379-386. doi:10.1002/pmrj.12424
13. Nissen M, Slim S, Jäger K, et al. Heart Rate Measurement Accuracy of Fitbit Charge 4 and Samsung Galaxy Watch Active2: Device Evaluation Study. *JMIR Form Res*. 2022;6(3):e33635. doi:10.2196/33635
14. Rodrigues E, Lima D, Barbosa P, et al. HRV Monitoring Using Commercial Wearable Devices as a Health Indicator for Older Persons during the Pandemic. *Sensors (Basel)*. 2022;22(5):2001. doi:10.3390/s22052001
15. Sjöberg V, Westergren J, Monnier A, et al. Wrist-Worn Activity Trackers in Laboratory and Free-Living Settings for Patients With Chronic Pain: Criterion Validity Study. *JMIR Mhealth Uhealth*. 2021;9(1):e24806. doi:10.2196/24806
16. Budig M, Keiner M, Stoohs R, Hoffmeister M, Höltke V. Heart Rate and Distance Measurement of Two Multisport Activity Trackers and a Cellphone App in Different Sports: A Cross-Sectional Validation and Comparison Field Study. *Sensors (Basel)*. 2021;22(1)doi:10.3390/s22010180
17. Boudreaux BD, Hebert EP, Hollander DB, et al. Validity of Wearable Activity Monitors during Cycling and Resistance Exercise. *Med Sci Sports Exerc*. Mar 2018;50(3):624-633. doi:10.1249/MSS.0000000000001471
18. Miranda Hurtado M, Reyes Vasquez J, Rodriguez-Fernandez M. Comparison of a tonometric with an oscillometric blood pressure monitoring device over 24 hours of ambulatory use. *Blood Press Monit*. 2021;26(2):149-155. doi:10.1097/mbp.0000000000000511
19. Luštrek M, Bohanec M, Cavero Barca C, et al. A Personal Health System for Self-Management of Congestive Heart Failure (HeartMan): Development, Technical Evaluation, and Proof-of-Concept Randomized Controlled Trial. *JMIR Med Inform*. 2021;9(3):e24501. doi:10.2196/24501
20. Villar R, Beltrame T, Hughson RL. Validation of the Hexoskin wearable vest during lying, sitting, standing, and walking activities. *Appl Physiol Nutr Metab*. Oct 2015;40(10):1019-24. doi:10.1139/apnm-2015-0140

21. Sayer G, Piper G, Vorovich E, et al. Continuous Monitoring of Blood Pressure Using a Wrist-Worn Cuffless Device. *Am J Hypertens*. 2022;35(5):407–413. doi:10.1093/ajh/hpac020
22. Kario K, Shimbo D, Tomitani N, Kanegae H, Schwartz JE, Williams B. The first study comparing a wearable watch-type blood pressure monitor with a conventional ambulatory blood pressure monitor on in-office and out-of-office settings. *J Clin Hypertens (Greenwich)*. 2020;22(2):135-141. doi:10.1111/jch.13799
23. Kinnunen H, Rantanen A, Kenttä T, Koskimäki H. Feasible assessment of recovery and cardiovascular health: accuracy of nocturnal HR and HRV assessed via ring PPG in comparison to medical grade ECG. *Physiol Meas*. 2020;41(4):04NT01. doi:10.1088/1361-6579/ab840a
24. Muggeridge DJ, Hickson K, Davies AV, et al. Measurement of Heart Rate Using the Polar OH1 and Fitbit Charge 3 Wearable Devices in Healthy Adults During Light, Moderate, Vigorous, and Sprint-Based Exercise: Validation Study. *JMIR Mhealth Uhealth*. 2021;9(3):e25313. doi:10.2196/25313
25. Climstein M, Alder JL, Brooker AM, et al. Reliability of the Polar Vantage M Sports Watch when Measuring Heart Rate at Different Treadmill Exercise Intensities. *Sports (Basel)*. 2020;8(9):117. doi:10.3390/sports8090117
26. Huang Q, Crumley T, Walters C, et al. "In-House" Data on the Outside-A Mobile Health Approach. *Clinical Pharmacology and Therapeutics*. 2020;107(4):948–956. doi:10.1002/cpt.1790
27. Ahn JH, Song J, Choi I, Youn J, Cho JW. Validation of Blood Pressure Measurement Using a Smartwatch in Patients With Parkinson's Disease. *Front Neurol*. 2021;12:650929. doi:10.3389/fneur.2021.650929
28. Burns A, Doheny EP, Greene BR, et al. SHIMMER: an extensible platform for physiological signal capture. *Annu Int Conf IEEE Eng Med Biol Soc*. 2010;2010:3759-62. doi:10.1109/IEMBS.2010.5627535
29. Morgado Areia C, Santos M, Vollam S, et al. A Chest Patch for Continuous Vital Sign Monitoring: Clinical Validation Study During Movement and Controlled Hypoxia. *J Med Internet Res*. 2021;23(9):e27547. doi:10.2196/27547
30. Berryhill S, Morton CJ, Dean A, et al. Effect of wearables on sleep in healthy individuals: a randomized crossover trial and validation study. *Journal of Clinical Sleep Medicine*. 2020;16(5):775–783. doi:10.5664/jcsm.8356
31. Jachymek M, Jachymek MT, Kiedrowicz RM, Kaźmierczak J, Płońska-Gościniak E, Peregud-Pogorzelska M. Wristbands in Home-Based Rehabilitation-Validation of Heart Rate Measurement. *Sensors (Basel)*. 2021;22(1):60. doi:10.3390/s22010060
32. Dolezal BA, Boland DM, Carney J, Abrazado M, Smith DL, Cooper CB. Validation of heart rate derived from a physiological status monitor-embedded compression shirt against criterion ECG. *J Occup Environ Hyg*. 2014;11(12):833-9. doi:10.1080/15459624.2014.925114
33. Mishra V, Pope G, Lord S, et al. Continuous Detection of Physiological Stress with Commodity Hardware. *ACM Trans Comput Healthc*. 2020;1(2):8:1–8:30. doi:10.1145/3361562
34. Al-Naami B, Abu Owida H, Abu Mallouh M, Al-Naimat F, Agha M, Al-Hinnawi AR. A New Prototype of Smart Wearable Monitoring System Solution for Alzheimer's Patients. *Med Devices (Auckl)*. 2021;14:423-433. doi:10.2147/mder.S339855
35. Mena LJ, Félix VG, Ostos R, et al. Mobile Personal Health Care System for Noninvasive, Pervasive, and Continuous Blood Pressure Monitoring: Development and Usability Study. *JMIR Mhealth Uhealth*. 2020;8(7):e18012. doi:10.2196/18012
36. Aktiia. Aktiia Healthcare Clinical Research Solutions. Accessed May 30, 2023, https://healthcare.aktiia.com/clinical-research/
37. Apple. Healthcare: Products and Platform. Accessed May 30, 2023, https://www.apple.com/sg/healthcare/products-platform/
38. Biobeat. Biobeat Products. Accessed May 30, 2023, https://www.bio-beat.com/products
39. Biofourmis. Biofourmis For Life Sciences. Accessed May 30, 2023, https://www.biofourmis.com/life-sciences
40. CardiacSense. Our Products: The Watch. Accessed May 30, 2023, https://www.cardiacsense.com/heart-rate-monitor-watch/
41. Cardioclogic. CardiacSense Medical Watch. Accessed October 20, 2023, https://www.cardiologic.co.uk/cardiacsense-watch
42. Corsano. Research – The Cardiowatch Bracelet Accessed May 30, 2023, https://corsano.com/research/
43. Corsano. Knowledge Base - Help Topics. Accessed October 9, 2023, https://corsano.com/knowledge-base/
44. Firstbeat. Firstbeat for Research. Accessed May 30, 2023, https://www.firstbeat.com/en/research-options/
45. Fitbit. Trackers — fitbit charge 6. Accessed October 9, 2023, https://www.fitbit.com/global/us/products/trackers/charge6

46. Fitbit. Health Solutions – Researchers FAQ. Accessed June 15, 2023, https://healthsolutions.fitbit.com/researchers/faqs/
47. Garmin. Products – Wearables – vívosmart 5. Accessed June 13, 2023, https://www.garmin.com.sg/products/wearables/vivosmart-5-mint/
48. Reddy RK, Pooni R, Zaharieva DP, et al. Accuracy of Wrist-Worn Activity Monitors During Common Daily Physical Activities and Types of Structured Exercise: Evaluation Study. *JMIR Mhealth Uhealth*. Dec 10 2018;6(12):e10338. doi:10.2196/10338
49. Garmin. Health – Business Solutions – Research. Accessed June 13, 2023, https://www.garmin.com/en-SG/health/business-solutions/research/
50. Hexoskin. Health Research & Professional Solutions Accessed May 30, 2023, https://www.hexoskin.com/pages/health-research
51. LiveMetric. LiveOne Band. Accessed June 7, 2023, https://livemetric.com/
52. Omron. HeartGuide(TM). Accessed June 7, 2023, https://omronhealthcare.com/products/heartguide-wearable-blood-pressure-monitor-bp8000m/
53. Ōura. Oura RIng Gen3. Accessed June 7, 2023, https://ouraring.com/product/heritage-gold
54. Polar. Polar H10 Heart Rate Sensor. Accessed June 7, 2023, https://www.polar.com/sg-en/sensors/h10-heart-rate-sensor
55. Polar. Vantage M2. Accessed June 6, 2023, https://support.polar.com/sg-en/vantage-m2
56. Samsung. Galaxy Watch6 Bluetooth (40mm). Accessed October 10, 2023, https://www.samsung.com/us/watches/galaxy-watch6/
57. VitalConnect. Patients – VitalPatch Frequently Asked Questions (FAQs). Accessed June 6, 2023, https://vitalconnect.com/patients/faqs/#faq-vitalpatch
58. WHOOP. Experience. Accessed June 15, 2023, https://www.whoop.com/experience/
59. Zephyr. Zephyr Performance Systems: Frequently Asked Questions. Accessed June 5, 2023, https://www.zephyranywhere.com/faqs
